# Older caregivers’ depressive symptomatology over time: evidence from the Survey of Health, Ageing and Retirement in Europe

**DOI:** 10.1007/s10433-024-00816-y

**Published:** 2024-07-19

**Authors:** Marie Agapitos, Graciela Muniz-Terrera, Annie Robitaille

**Affiliations:** 1 Département de Psychologie, Université du Québec à Montréal, Montreal, QC Canada; 2 Ohio University (Heritage College of Osteopathic Medicine), Athens, OH USA; 3 University of Edinburgh (Edinburgh Dementia Prevention), Edinburgh, Scotland; 4 University of Ottawa (Interdisciplinary School of Health Sciences), Ottawa, ON Canada; 5 Perley Health (Centre of Excellence in Frailty-Informed Care), Ottawa, ON Canada

**Keywords:** Caregiving, Longitudinal, Depressive symptoms, Older adults

## Abstract

The prevalence of informal caregiving is increasing as populations across the world age. Caregiving has been found to be associated with poor mental health outcomes including depressive symptoms. The purpose of this study is to examine the mean trajectory of depressive symptomatology in older caregivers in a large European sample over an eight-year period, the effects of time-varying and time-invariant covariates on this trajectory, and the mean trajectory of depressive symptomatology according to pattern of caregiving. The results suggest that depressive symptoms in the full sample of caregivers follow a nonlinear trajectory characterized by an initial decrease which decelerates over time. Caregiver status and depressive symptoms were significantly associated such that depressive symptoms increased as a function of caregiver status. The trajectory in caregivers who report intermittent or consecutive occasions of caregiving remained stable over time. Significant associations were found between sociodemographic, health and caregiving characteristics and the initial levels and rates of change of these trajectories. While these results point to the resilience of caregivers, they also highlight the factors that are related to caregivers’ adaptation over time. This can help in identifying individuals who may require greater supports and, in turn, ensuring that caregivers preserve their well-being.

## Introduction

As populations across the world continue to age at an accelerating rate, the proportion of older adults is growing more rapidly than any other group (United Nations 2015). With this, there is an increase in the proportion of the population living with complex, chronic and often comorbid conditions (Prince et al. 2015). These conditions, whether physical or cognitive, may involve longer or more intense periods of disability and care dependence (Prince et al. 2015) which could entail a strong reliance on both formal healthcare structures and on unpaid caregivers (United Nations 2015). Indeed, caregivers are considered to be an integral part of care provision to older adults, often filling in the gaps inherent to formal healthcare systems (Bookman and Harrington 2007). Those individuals providing unpaid care to these older adults are most often spouses or adult children, and they are often older adults themselves (Agree and Glaser 2009).

The provision of care to a loved one is considered by many to be a physically and psychologically demanding activity (Schulz et al. 2020). Caregivers may be responsible for the provision of assistance with a broad range of care tasks. This can include instrumental activities of daily living, which involve tasks such as managing household finances, managing and dispensing medications, and meal preparation, and activities of daily living, which involve assistance with personal care tasks such as bathing, dressing and feeding (Roth et al. 2015; Mlinac and Feng 2016). Beyond this, caregivers are often actively engaged in care planning, advocacy, and social and emotional support for their loved one (Wittenberg et al. 2018). For many, this role may be maintained for several years, with the nature and intensity of their responsibilities evolving with their loved ones’ condition (Roth et al. 2015).

The breadth, depth and duration of responsibilities associated with providing care to older adults may place caregivers at an increased risk of negative mental health outcomes (Pinquart and Sörensen 2003; Sambasivam et al. 2019) including stress and burden (e.g., Kim et al. 2012; Adelman et al. 2014), depressive and anxious symptomatology or diagnosed episodes (Schulz and Sherwood 2008), and impaired quality of life (Schulz and Sherwood 2008). Depressive symptoms are among the most prevalent and persistent outcomes associated with caregiving (Choi and Bohman 2007)*.* Indeed, numerous cross-sectional studies have demonstrated higher rates of depressive symptoms and depression among caregivers as compared to non-caregivers (Navaie-Waliser et al. 2002; Pinquart and Sörensen 2003, Cuijpers 2005; del-Pino-Casado, Palomino-Moral et al. 2017; Loh et al. 2017; Collins and Kishita 2019; del-Pino-Casado, Rodríguez Cardosa et al. 2019).

While these cross-sectional studies provide a portrait of the caregiving role and the prevalence of mental health outcomes such as depression, they do not consider the fact that caregiving is a dynamic process rather than a fixed experience (Aneshensel et al. 1995). The nature and extent of caregivers’ responsibilities may vary over time in accordance with changes in the care recipients’ health status and level of care dependence (Roth et al. 2015). In turn, their experiences of reactions to these changes may also evolve (Aneshensel et al. 1995). Through their stress process model, Pearlin et al. (1990) posit that stressors proliferate over the course of caregiving and thus contribute to poorer outcomes. Their model suggests that, coupled with individual contextual factors (e.g., sociodemographic characteristics and characteristics of the caregiving situation), the stress directly associated with the provision of care may eventually result in stress in other areas of life (Pearlin et al. 1990). The literature suggests that this stress process may follow different pathways, one described as the wear and tear hypothesis and the other as the adaptation hypothesis (Helson 1964; Townsend et al. 1989). The wear and tear hypothesis posits that the cumulative effect of these stressors leads to the worsening of caregivers’ mental health over time (Townsend et al. 1989), whereas the adaptation hypothesis suggests that caregivers may adapt to their role and experience an improvement in mental health outcomes over time (Helson 1964).

Considering this dynamic nature of caregiving and its associated outcomes, a growing body of literature has sought to examine mean trajectories of depressive symptoms or psychological distress over time among current caregivers (Roth et al. 2001; Romero-Moreno et al. 2012; Borsje et al. 2016; Lacey et al. 2019). The results of these studies most often suggest that mental health outcomes (depressive symptoms or psychological distress) among caregivers follow a stable trajectory (Roth et al. 2001; Romero-Moreno et al. 2012; Borsje et al. 2016; Lacey et al. 2019). Others have found that depressive symptomatology increases over time (Bookwala 2009; Barnett 2015). Bookwala (2009) identified this particular trajectory of worsening depressive symptoms among women but found a trajectory of improvement in depressive symptoms over time among men. These studies found higher initial levels of symptomatology among caregivers who were women (Borsje et al. 2016; Lacey et al. 2019), were younger in age (Roth et al. 2001; Borsje et al. 2016), reported lower socioeconomic status (Roth et al. 2001; Barnett 2015) and lesser educational attainment (Barnett 2015), provided care to a spouse (Roth et al. 2001; Borsje et al. 2016), reported longer duration of caregiving in years or intermittent patterns of caregiving (Lacey et al. 2019), and whose care recipients presented with higher levels of neuropsychiatric symptoms (Borsje et al. 2016). These results suggest that some caregivers are more likely to experience a wear and tear process associated with caregiving (Bookwala 2009; Barnett 2015), others are more likely to adapt (Bookwala 2009), and still others experience neither wear and tear nor adaptation processes (Roth et al. 2001; Romero-Moreno et al. 2012; Borsje et al. 2016; Lacey et al. 2019).

The present study aims to build upon existing knowledge on the long-term experiences of caregivers and the factors influencing their depressive symptomatology. Employing an advanced statistical analysis on a large, cross-national sample over a longer period of time improves the likelihood of capturing the long-term evolution of the caregiver role and its associated symptomatology (Roth et al. 2015) and represents an important contribution. Thus, this study aims to (1) examine the mean course of depressive symptomatology in older caregivers in a large European sample over an eight-year period, (2) examine whether change in caregiver status at each time point is related to change in depressive symptomatology at the same time point, (3) examine the effects of sociodemographic, health-related and caregiving variables on participating caregivers’ initial levels of depressive symptoms and the rate and shape of the change in these symptoms over time and (4) examine the mean course of depressive symptomatology according to different patterns of care provision (single occasion, intermittent and two or more consecutive occasions).

## Method

### Data and sample

Data for this study originated from the Survey of Health, Ageing and Retirement in Europe (SHARE Börsch-Supan, Brandt et al. 2013). SHARE is a multidisciplinary, cross-national panel study of adults aged 50 years or over with bi-annual data collection waves beginning in 2004 (Börsch-Supan, Brandt et al. 2013). A total of 30,424 participants from twelve countries were included in SHARE at baseline with a further seventeen countries and refreshment samples joining at subsequent waves (Börsch-Supan, Brandt et al. 2013). Data are collected on a wide variety of modules including but not limited to demographics characteristics, physical, cognitive and mental health, and employment (Börsch-Supan, Brandt et al. 2013).

Seven waves of data were available at the time of study design, data preparation and analysis. Countries with two or more waves of data at wave 7 of SHARE were included in the study sample (Austria, Germany, Sweden, Netherlands, Spain, Italy, France, Denmark, Greece, Switzerland, Belgium, Israel, Czech Republic, Poland, Luxembourg, Hungary, Portugal, Slovenia, Estonia, Croatia). The third wave of the study (SHARELIFE) focused on participants’ life histories, and the regular panel interview was not administered (Börsch-Supan, Brandt et al. 2013) and was missing key study variables (e.g., caregiving variables, depressive symptoms). As such, it was not included in the present analysis. Therefore, our sample included data from SHARE waves 1, 2, 4, 5, 6 and 7 (SHARE-ERIC 2020a, b, c, d, e, f).

From this sample, participants were selected based on caregiver status and characteristics of the person to whom they were providing care. SHARE identifies caregivers through two questions*: Is there someone living in this household whom you have helped regularly during the last twelve months with personal care, such as washing, getting out of bed, or dressing?* and *In the last twelve months, have you personally given any kind of help listed on card 28 (personal care, practical household help, help with paperwork) to a family member from outside the household, a friend or neighbor?* Participants who responded Yes to one or both questions at any wave of SHARE were selected. From these, individuals who reported providing care to older adults (spouse, parent, or other relatives, neighbor or friend) were included in the study sample (*N* = 16 412). Those who reported providing care to grandchildren or other children were not included. Participants who were aged younger than 50 at baseline (*N* = 527), with missing data on covariates or outcome variable at baseline (*N* = 1 898), were excluded.

### Data organization

Because our sample included participants who endorsed caregiving at any point during the observation period and our aim was to examine depressive symptoms in caregivers, we reorganized the data such that participants were included as of their first instance of caregiving. Individual participants’ data were realigned to a common baseline, henceforth referred to as Time 1. This realignment resulted in a data file in which all participants reported providing care to an older adult at Time 1. For example, a participant who reported caregiving for the first time at study wave 4 was included in our sample as of that wave (wave 4 would become Time 1). Participants were kept in the sample at subsequent time points regardless of whether they were still caregiving to capture the intermittent nature and long-term impact of caregiving. Once this process was complete, few caregivers remained at Time 5 and 6 (*N* = 251 and *N* = 82, respectively) and these time points were not included in the final model.

## Measures

### Depressive symptoms

Depressive symptoms were measured using the EURO-D Geriatric Depression Scale, a measure of late-life depression (Prince et al. 1999). The EURO-D assesses the presence or absence of twelve depressive symptoms: depressed mood, pessimism, suicidality, guilt, sleep disturbance, loss of interest, irritability, change in appetite, fatigue, difficulty concentrating, loss of enjoyment and tearfulness (Prince et al. 1999). Each item is scored as 1 (symptom present over the last month) or 0 (symptom absent over the last month), with total scores ranging from 0 (not depressed) to 12 (very depressed). The EURO-D was validated in EURODEP, a European study of depression prevalence (Prince et al. 1999, 2015) as well as in SHARE (Castro-Costa, Dewey et al. 2008). This scale was found to have both adequate internal consistency and criterion validity when compared to other measures of depression (Prince et al. 1999; Guerra et al. 2015).

### Covariates

The following variables were included in the model as time-invariant covariates on the intercept and slopes: age, sex, marital status, years of education, employment status, self-perceived health, number of chronic conditions and relationship to the care recipient. To facilitate the interpretation of results, continuous covariates (age, years of education and number of chronic conditions) were centered on their means. Sex was coded as 0 (male) or 1 (female). The remaining categorical covariates were coded as binary variables. Marital status was coded as 0 (separated, divorced, widowed) or 1 (married or registered partnership). Employment status was coded as 0 (not employed or retired) or 1 (employed or self-employed). Self-perceived health was coded as 0 (fair or poor) or 1 (excellent, very good, good). Because relationship of the care recipient to the caregiver included three categories (spouse, parent or other, where other includes relatives, friends or neighbors), it was recoded into two binary variables with spouse as the reference category. Spouse was coded as 0, and parent or other was coded as 1. Location of care provision (inside the home or outside the home) was highly correlated with relationship to the care recipient (r − 0.803) suggesting that multicollinearity exists and was thus not included in the present analysis.

The decision to include variables which do or may vary over time as time invariant is a methodological one. Because age is perfectly correlated with the time variable included in our model, it was centered on the mean and included as time invariant. While other covariates may vary over time (e.g., a participant’s marital status or self-perceived health may change over the course of the study), the inclusion of multiple covariates as time varying complexifies the statistical model considerably. Thus, it was decided to control for these covariates at baseline.

Caregiver status at each time point was coded as 0 (not a caregiver) or 1 (caregiver). This variable was then used in two different ways. In our first model examining the mean trajectory for all caregivers, it was included as a time-varying covariate to assess the association between caregiver status on level of depressive symptomatology at each time point. For subsequent models, we computed a variable for caregiving pattern which considered three possible patterns: those with a single occasion of caregiving, those with intermittent or on-and-off occasions of caregiving and those with two or more consecutive occasions of caregiving. This variable was used to create distinct samples of data per caregiving status on which analyses were conducted. Patterns of caregiving status at each time point are reported in Table S5 in appendix.

### Statistical analysis

**Fig. 1:**
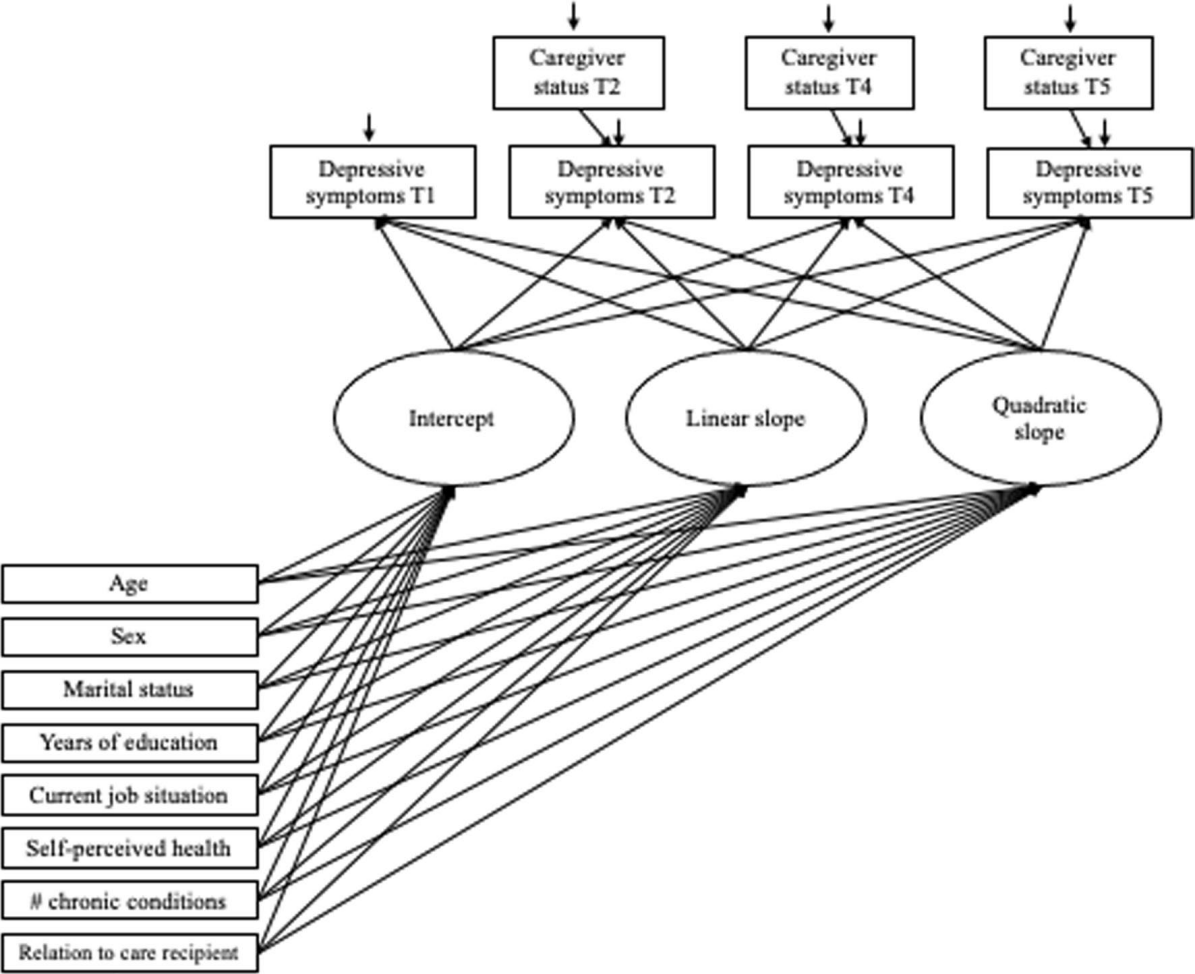
Path diagram for the conditional latent growth curve model including caregiver status as a time-varying covariate

**Fig. 2:**
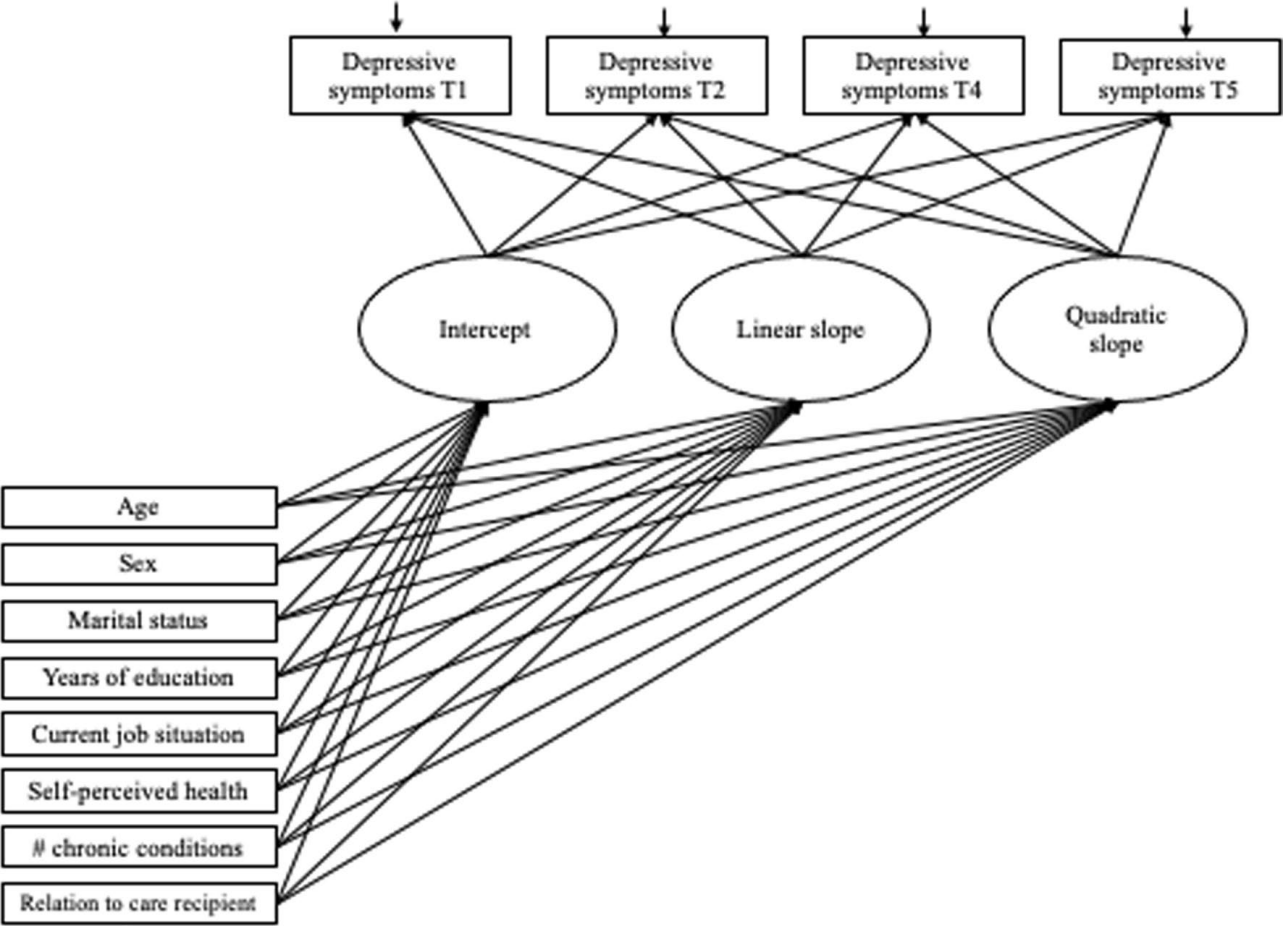
Path diagram for the conditional latent growth curve models fitted for each pattern of caregiving

Conditional latent growth curve models (LGM) were fitted to the data. The first model included all participants, and caregiver status was included as a time-varying covariate. The path diagram for the first LGM is presented in Fig. 1. The next models were fitted to subsamples of data according to caregiving pattern (single occasion, intermittent, two or more occasions). Because caregiver status was accounted for through the pattern of caregiving, it was not included as a time-varying covariate in these models. The path diagram for the LGM fitted to pattern-specific subsamples of data is presented in Fig. 2.

Fixed effects and random effects models were estimated to determine the mean growth curve of depressive symptoms and to assess the extent of between-person variability around the mean curve (Curran et al. 2010). Depressive symptom scores were modeled as a function of years in the study. Both linear and quadratic models were fitted. Levels of depressive symptoms and rates of change were evaluated in association with the study covariates. Residual variances were fixed to be equal across time points. Akaike Information Criterion (AIC) and Adjusted Bayesian Information Criterion (Adjusted BIC) were examined to assess both linear and quadratic model fit and to inform model selection. The LGM was run using MPlus version 8.4 (Muthén and Muthén 1998–2017). A Missing Value Analysis conducted using SPSS Version 27 revealed that participants missing at Times 2, 3 and 4 were significantly older and reported having significantly more chronic conditions. Participants missing at Time 4 had significantly higher educational attainment in years. Given that the data were not MCAR, missing data were addressed in Mplus using Full Information Maximum Likelihood (FIML). Maximum likelihood robust (MLR) estimation was used to obtain parameter estimates. A standard assumption of growth modeling, including LGM, is that the random effects are normally distributed. Mplus uses FIML and MLR to take into account possible non-normality and non-independence of observations (Muthén and Muthén 1998–2024).

## Results

Characteristics of study participants at Time 1 are reported in Table 1. Number of participants, number of caregivers, and means and standard deviations for depressive symptom scores are reported in Table 2. Individual trajectories of depressive symptoms for a randomly selected subset of the study sample (*N* = 200) are graphically depicted in Fig. 5 in appendix.

**Table 1:** Participant characteristics at Time 1 (*N* = 13,987)

	%
Sex, female	61.26
Marital status, partnered	85.01
Employment status, employed	26.28
Self-perceived health, excellent-good	57.33
*Care recipient relationship*	
Spouse	58.20
Parent	12.53
Other	29.27
*Caregiving pattern*	
Single occasion	80.17
Intermittent occasions	4.73
Two or more occasions	15.10

	*M* (SD)
Age, years	65.53 (9.75)
Education, years	10.77 (4.34)
Number of chronic conditions	1.24 (1.27)

Participants’ first instance of caregiving in the study was realigned to a new baseline (Time 1)

Data source: SHARE Release 7.1.0

**Table 2:** Depressive symptom scores at each time point

	Time 1	Time 2	Time 3	Time 4
EURO-D M(SD)	3.02 (2.42)	2.82 (2.41)	2.78 (2.39)	2.67 (2.38)
*N(% data present)*				
Total respondents	13,987	6848 (48.96)	3833 (27.40)	2491 (17.81)
Caregivers	13,987	2172 (15.53)	770 (5.51)	375 (2.68)

EURO-D = Scores on the EURO-D Geriatric Depression Scale. Participants were included in our analysis as of their first instance of caregiving, regardless of future caregiver status. As such, the total number of respondents to the EURO-D variables and the number of caregivers at each wave are not equivalent. Both values are presented above. Participants’ first instance of caregiving in the study was realigned to a new baseline (Time 1). Data source: SHARE Release 7.1.0

### LGM with caregiver status as a time-varying covariate

AIC and Adjusted BIC values were compared for linear and quadratic models. They are presented in Table S6 in appendix. The quadratic model had lower values on both AIC and Adjusted BIC as compared to the linear model which suggests better fit. The quadratic model was thus selected.

**Fig. 3:**
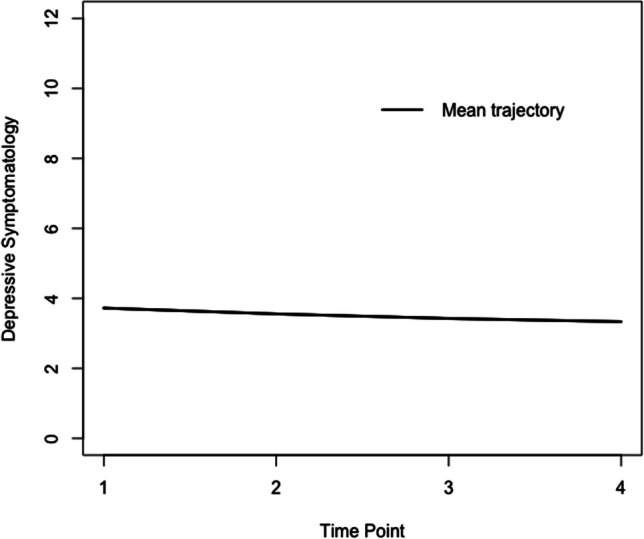
Mean trajectory of depressive symptomatology

Estimates for the mean trajectory of depressive symptoms and effects of covariates on the intercept and slopes are presented in Table 3. On average, participating caregivers had a score of 3.93 on the EURO-D at Time 1, with the linear slope estimated as − 0.225 (SE = 0.049) and the quadratic slope estimated as 0.019 (SE = 0.006). These suggest that the mean trajectory is characterized by a significant decrease in depressive symptoms which decelerates over time. The mean trajectory is depicted graphically in Fig. 3.

**Table 3:** Estimates for the mean trajectory of depressive symptoms and effects of covariates on the intercept and slopes of depressive symptoms

	Estimate	*SE*	*p*
*Fixed effects*			
Intercept	3.930	0.091	0.000
Linear slope	− 0.225	0.049	0.000
Quadratic slope	0.019	0.006	0.004
*Random effects*			
Intercept	2.249	0.082	0.000
Linear slope	0.168	0.035	0.000
Quadratic slope	0.002	0.001	0.000
*Intercept*			
Age	− 0.008	0.003	0.001
Sex	0.865	0.038	0.000
Marital status	− 0.340	0.053	0.000
Education	− 0.044	0.005	0.000
Employment	− 0.174	0.049	0.000
Chronic conditions	0.199	0.017	0.000
Self-perceived health	− 1.588	0.043	0.000
Parent care recipient	− 0.308	0.060	0.000
Other care recipient	− 0.293	0.047	0.000
*Linear slope*			
Age	0.008	0.002	0.001
Sex	− 0.051	0.027	0.065
Marital status	0.061	0.038	0.106
Education	− 0.003	0.003	0.330
Employment	0.020	0.034	0.550
Chronic conditions	0.005	0.013	0.681
Self-perceived health	0.187	0.031	0.000
Parent care recipient	− 0.007	0.043	0.864
Other care recipient	− 0.010	0.034	0.759
*Quadratic slope*			
Age	− 0.001	0.000	0.028
Sex	0.004	0.004	0.293
Marital status	− 0.002	0.005	0.693
Education	0.001	0.000	0.227
Employment	− 0.003	0.004	0.563
Chronic conditions	− 0.001	0.002	0.633
Self-perceived health	− 0.015	0.002	0.000
Parent	0.000	0.006	0.939
Other	0.000	0.004	0.915
*Time-varying covariate*			
Caregiver status	0.243	0.054	0.000

SE refers to the standard error of the parameter estimates, and p refers the two-tailed p-value. Data source: SHARE Release 7.1.0

Caregivers who were older at Time 1 compared to those who were younger, caregivers who were married or partnered compared to those who were single, divorced, separated or widowed, caregivers who had completed more years of education compared to those who had completed less, caregivers who were currently employed compared to those who were not, caregivers who reported greater self-perceived health compared to those who reported poorer self-perceived health and those who reported caring for a parent or other care recipient compared to those who reported caring for a spouse experienced lower levels of depressive symptoms at Time 1. Caregivers who were women compared to men and caregivers who reported a higher number of chronic conditions compared to those who reported fewer experienced higher levels of depressive symptoms at Time 1.

Caregivers who were older at Time 1 compared to those who were younger and caregivers who reported greater self-perceived health compared to those who reported poorer self-perceived health demonstrated a positive change in the linear slope of depressive symptoms (flatter decrease) and a negative change in the quadratic slope of depressive symptoms (slower deceleration). Sex, marital status, educational attainment, employment status, number of chronic conditions and relationship to the care recipient were not significantly associated with the linear or quadratic slopes of depressive symptoms.

After controlling for growth factors, a significant within-person association was found between caregiver status and depressive symptoms whereby depressive symptoms increased as a function of caregiver status. On each measurement occasion, identification as a caregiver was associated with a higher score on depressive symptoms.

### LGM per caregiving pattern

**Fig. 4:**
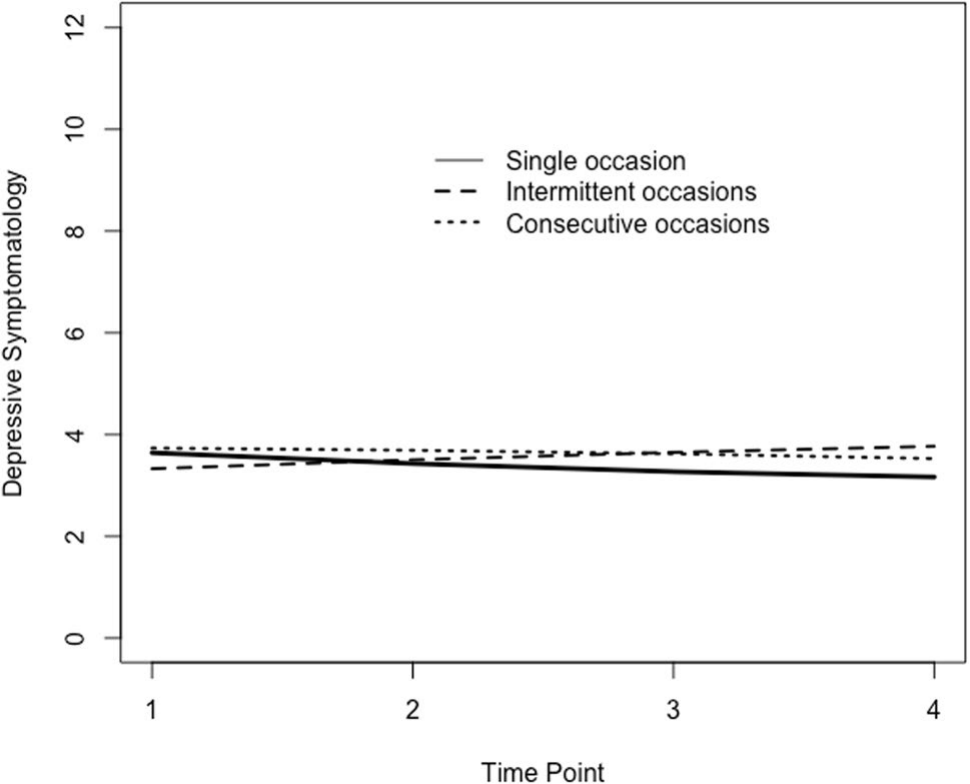
Mean trajectories of depressive symptomatology by caregiving pattern

**Fig. 5:**
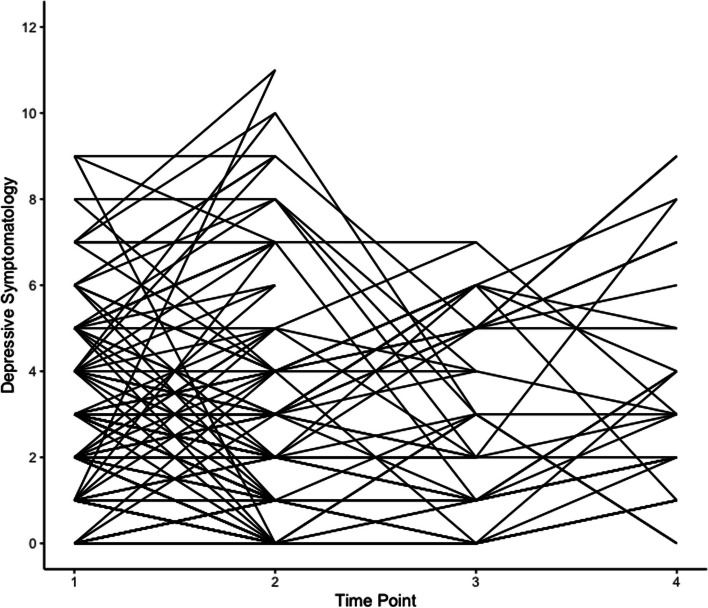
Individual trajectories of depressive symptoms for a random subset of participants

AIC and Adjusted BIC values were compared for linear and quadratic models for all three caregiving patterns. They are presented in Table S6 in appendix. The quadratic model was thus selected on the basis of these fit indices and for ease of comparison with the full sample LGM described above. Estimates for the mean trajectory of depressive symptoms and effects of covariates on the intercept and slopes for all three models are presented in Table 4. The mean trajectories for each pattern of caregiving are presented in Fig. 4.

**Table 4:** Estimates for the mean trajectories of depressive symptoms and effects of covariates on the intercepts and slopes of depressive symptoms for each caregiving pattern

	Single instance of CG *N* = 11,284			Intermittent CG *N* = 670			Two or more instances of CG *N* = 2143		
	Estimate	*SE*	*p*	Estimate	*SE*	*p*	Estimate	*SE*	*p*
*Fixed effects*									
Intercept	3.909	0.075	0.000	3.125	0.000	0.000	3.751	0.177	0.000
Linear slope	− 0.294	0.059	0.000	0.217	0.155	0.155	− 0.004	0.098	0.969
Quadratic slope	0.027	0.008	0.001	− 0.014	0.508	0.508	− 0.013	0.012	0.293
*Random effects*									
Intercept	2.386	0.101	0.000	1.880	0.000	0.000	1.872	0.174	0.000
Linear slope	0.211	0.045	0.000	0.039	0.645	0.645	0.082	0.069	0.236
Quadratic slope	0.002	0.001	0.001	− 0.014	0.662	0.662	0.001	0.001	0.178
*Intercept*									
Age	− 0.007	0.003	0.011	− 0.205	0.013	0.050	− 0.012	0.007	0.069
Sex	0.872	0.042	0.000	0.860	0.178	0.000	0.806	0.095	0.000
Marital status	− 0.379	0.059	0.000	0.237	0.291	0.414	− 0.232	0.139	0.096
Education	− 0.042	0.005	0.000	− 0.025	0.021	0.225	− 0.057	0.012	0.000
Employment	− 0.163	0.055	0.003	− 0.497	0.213	0.020	− 0.148	0.120	0.216
Chronic conditions	0.197	0.019	0.000	0.117	0.076	0.124	0.229	0.043	0.000
Self-perceived health	− 1.595	0.048	0.000	− 1.564	0.188	0.000	− 1.569	0.104	0.000
Parent care recipient	− 0.338	0.070	0.000	0.186	0.229	0.417	− 0.317	0.135	0.019
Other care recipient	− 0.296	0.052	0.000	− 0.148	0.212	0.486	− 0.343	0.127	0.007
*Linear slope*									
Age	0.009	0.002	0.000	0.005	0.007	0.471	0.009	0.004	0.020
Sex	− 0.078	0.034	0.021	0.024	0.090	0.789	0.017	0.056	0.762
Marital status	0.133	0.045	0.003	− 0.238	0.127	0.061	− 0.092	0.080	0.248
Education	0.000	0.004	0.938	− 0.017	0.010	0.098	− 0.005	0.007	0.414
Employment	0.032	0.042	0.450	− 0.026	0.109	0.809	0.20	0.072	0.785
Chronic conditions	− 0.007	0.016	0.687	0.032	0.040	0.418	0.026	0.023	0.271
Self-perceived health	0.204	0.039	0.000	0.019	0.097	0.845	0.222	0.060	0.000
Parent care recipient	− 0.011	0.053	0.831	− 0.241	0.122	0.049	0.011	0.087	0.902
Other care recipient	− 0.033	0.040	0.405	0.141	0.105	0.178	− 0.061	0.075	0.414
*Quadratic slope*									
Age	− 0.01	0.000	0.052	0.000	0.001	0.674	− 0.001	0.001	0.060
Sex	0.007	0.004	0.110	− 0.013	0.012	0.270	0.001	0.007	0.927
Marital status	− 0.011	0.006	0.054	0.030	0.017	0.074	0.018	0.010	0.068
Education	0.000	0.001	0.754	0.001	0.001	0.300	0.001	0.001	0.237
Employment	− 0.003	0.006	0.535	− 0.002	0.015	0.919	0.000	0.009	0.958
Chronic conditions	0.000	0.002	0.925	− 0.002	0.005	0.741	− 0.004	0.003	0.258
Self-perceived health	− 0.017	0.005	0.001	0.006	0.013	0.632	− 0.022	0.008	0.008
Parent care recipient	0.000	0.007	0.997	0.028	0.017	0.096	− 0.002	0.012	0.897
Other care recipient	0.003	0.005	0.596	− 0.015	0.013	0.251	0.004	0.010	0.694

CG = caregiving, SE = the standard error of the parameter estimates, and *p* = the two-tailed *p*-value. Data source: SHARE Release 7.1.0

#### Single occasion of caregiving

On average, caregivers who reported a single occasion of caregiving had a score of 3.91 on the EURO-D at Time 1, with the linear slope estimated as − 0.294 (SE = 0.059) and the quadratic slope estimated as 0.027 (SE = 0.008). These suggest that the mean trajectory for participants with a single instance of caregiving is characterized by a significant decrease in depressive symptoms which decelerates over time.

Among caregivers with a single instance of caregiving, lower initial levels of depressive symptoms were found among those who were older at Time 1 compared to those who were younger, those who were married or partnered compared to those who were single, divorced separated or widowed, those who had completed more years of education compared to those who had completed fewer, those who were currently employed compared to those who were not and those who reported providing care to a parent or other care recipient compared to those who reported providing care to a spouse. Higher initial levels of depressive symptoms were found among caregivers who were women compared to those who were men and those who reported more chronic conditions compared to those who reported fewer.

Among caregivers with a single instance of caregiving, those who were older at Time 1 compared to those who were younger, those who were married or partnered compared to those who were single, divorced or widowed demonstrated a positive change in the linear slope of depressive symptoms, and those who were women compared to those who were men demonstrated a negative linear slope in depressive symptoms over time. Those who reported greater self-perceived health compared to those who reported poorer self-perceived health demonstrated a positive change in the linear slope of depressive symptoms and a negative change in the quadratic slope of depressive symptoms. Educational attainment, employment, chronic conditions and relationship to the care recipient were not significantly associated with the linear slope of depressive symptoms. Age, sex, marital status, education, employment, chronic conditions and relationship to the care recipient were not significantly associated with the quadratic slope of depressive symptoms.

#### Intermittent instances of caregiving

On average, caregivers who reported intermittent occasions of caregiving had a score of 3.13 on the EURO-D at Time 1, with the linear slope estimated as 0.217 (SE = 0.155) and the quadratic slope estimated as − 0.014 (SE = 0.508). These suggest that the mean trajectory for participants with intermittent instances of caregiving is characterized by depressive symptoms which are stable over time.

Among caregivers with intermittent occasions of caregiving, lower initial levels of depressive symptoms were found in those who were older at Time 1 compared to those who were younger, those who were currently employed compared to those who were not, and those who reported greater self-reported health compared to those who reported poorer self-reported health. Higher initial levels of depressive symptoms were found in caregivers who were women compared to those who were men. None of the study covariates were significantly associated with the linear or quadratic slopes of depressive symptoms.

#### Two or more consecutive occasions of caregiving

On average, caregivers who reported two or more consecutive waves of caregiving had a score of 3.75 on the EURO-D at Time 1, with the linear slope estimated as − 0.004 (SE = 0.098) and the quadratic slope estimated as − 0.013 (SE = 0.012). These suggest that the mean trajectory for caregivers with consecutive occasions of caregiving is characterized by depressive symptoms which are stable over time.

Among caregivers who reported two or more consecutive occasions of caregiving, lower initial levels of depressive symptoms were observed in those who had completed more years of education compared to those who had completed fewer, those who reported greater self-perceived health compared to those who reported poorer self-perceived health and those who reported providing care to a parent compared to those who reported providing care to a spouse. Higher initial levels of depressive symptoms were observed in those who were women compared to men and those who reported more chronic conditions compared to those who reported fewer.

Among caregivers who reported two or more consecutive instances of caregiving, those who were older at Time 1 compared to those who were younger and those who reported greater self-perceived health compared to those who reported poorer self-perceived health demonstrated a positive linear slope of depressive symptoms. Sex, marital status, education, employment, chronic conditions and relationship to the care recipient were not significantly associated with the linear slope of depressive symptoms, and none of the study covariates were significantly associated with the quadratic slope of depressive symptoms.

## Discussion

Considering the current and growing prevalence of caregiving, understanding whether and how caregivers’ mental health evolves over time is not only timely but essential. Studies such as SHARE (Börsch-Supan, Brandt et al. 2013), which collect individual-level, longitudinal data in a cross-national setting, provide a unique opportunity to explore such trajectories. Thus, the present study aimed to characterize the course of depressive symptomatology over an eight-year period in a sample of older caregivers while also controlling for both time-varying and time-invariant covariates, and to characterize the course of depressive symptomatology according to caregiving pattern.

Our findings from the first model including all participating caregivers while controlling for caregiver status at each time point and baseline characteristics including but not limited to age, sex and marital status suggest that, on average, participating caregivers’ depressive symptoms at Time 1 were nearing clinical significance. Our findings from caregiving pattern-specific models found similar levels of depressive symptoms at Time 1. Guerra et al. (2015) and Prince et al. (1999) suggest an optimal cut-point of 4 on the EURO-D Geriatric Depression Scale for the identification of depression. While the purpose of this study was not to identify diagnosable levels of depressive symptoms, this finding is consistent with the extensive body of literature which has reported high levels of depressive symptoms in caregivers (e.g., Pinquart and Sörensen 2003; del-Pino-Casado, Palomino-Moral et al. 2017; Collins and Kishita 2019).

Regarding the factors associated with initial levels of depressive symptoms, our results regarding the effect of sex, age, caregiving duration and relationship to the care recipient were consistent with previous longitudinal studies and the broader caregiving literature (Roth et al. 2001; Borsje et al. 2016; Lacey et al. 2019). Our results are consistent with previous studies which have found that spousal caregivers tend to experience higher levels of depressive symptoms (Roth et al. 2001; Pinquart and Sörensen 2011; Borsje et al. 2016). This was observed in our model including the full sample of caregivers, as well as the model for caregivers with two or more consecutive occasions of caregiving. Spousal caregiving is often considered to be more intensive than other forms of care. Cohabitation with the care recipient, as is more often the case with spousal caregivers, may translate to more hours of care provided and less respite for the caregivers (Pinquart and Sörensen 2011). The results from each of our models, whether for the full sample of caregivers or for different patterns of caregiving, are also consistent with those studies who have found higher levels of depressive symptoms among caregivers who are women (Borsje et al. 2016; Lacey et al. 2019). This has been attributed to the fact that women caregivers tend to provide more hours of care and provide more personal care than their male counterparts (Pinquart and Sörensen 2006).

The significant association between educational attainment and depressive symptoms was also consistent with current knowledge, providing further evidence for the fact that higher educational attainment tends to be associated with lower levels of depressive symptoms (Cohen et al. 2020). The significant associations between self-perceived health and depressive symptoms across all models and number of chronic conditions and depressive symptoms across all models with the exception of the intermittent caregiving model were also consistent with the caregiving literature. Interestingly, our results found that, while greater self-perceived health was indeed associated with lower depressive symptoms at Time 1, it was also associated with a slower decrease in depressive symptoms over time. This could suggest that greater self-perceived health may be a protective factor at Time 1, but that its impact is tempered over time. It has also been posited that individuals with better self-perceived health self-select into the caregiver role, and those with poorer self-perceived health may experience more distress with care provision and select out (Pinquart and Sörensen 2011). The association between depressive symptoms and self-perceived health provides further nuance to the interpretation of these results. Some have posited that the association between depressive symptoms and self-perceived health may constitute a confounding factor (De Frias et al. 2005); in a longitudinal context, continued presence of depressive symptoms over time may then influence scores on self-perceived health over time.

The finding that employment was associated with lower initial levels of depressive symptoms is also of interest. Models such as Pearlin et al.’s (1990) stress process model posit that the combination of paid employment and caregiving responsibilities may exacerbate stress, in turn leading to poorer mental health outcomes. However, our results across all models with the exception of the consecutive caregiving pattern model suggest that paid employment may be a protective factor, at least regarding initial levels of depressive symptoms. Explanations for this association have been advanced, notably that engagement in paid employment may provide caregivers with the opportunity to temporarily remove themselves from their role of caregiver and may increase access to a supportive social network (O’Neill et al. 2021).

This study also generated important findings regarding the shape and rate of change of depressive symptoms over time. Our results from the model including the full sample of caregivers point to a trajectory that is characterized by a decrease in depressive symptoms which decelerates over time. This is contrast with those studies who have found that depressive symptoms among caregivers tend to remain stable over time, regardless of whether initial levels are higher or lower (Roth et al. 2001; Romero-Moreno et al. 2012; Borsje et al. 2016; Lacey et al. 2019) and those who have found the inverse (Bookwala 2009; Barnett 2015). When considered through the lens of Townsend et al. (1989)’s wear and tear and adaptation hypotheses, these results point to an adaptation process which appears to attain a floor. This could speak to an accumulation of stressors which eventually outweigh caregivers’ capacity to cope and adapt (Townsend et al. 1989; Pearlin et al. 1990).

The inclusion of caregiver status as a time-varying covariate adds important nuance to these results. Participants were included in this sample as of the first occasion in which they identified as caregivers; however, they did not necessarily remain caregivers at each of the three subsequent time points (see Table 2). Examining trajectories of depressive symptoms in individuals who have provided care but have stopped, or who have provided care intermittently, remains valuable. The literature has found that negative impacts of care provision can both endure and evolve beyond the end of care provision (Schulz et al. 2020). The nature and intensity of these outcomes may vary with the reasons for the end of care provision (e.g., long-term care placement, death or other) and could involve relief and recovery or continued and worsening psychological morbidity (Schulz et al. 2020). The significant positive association between caregiving status and depressive symptoms provides further evidence that caregiving appears to be a risk factor for higher depressive symptomatology (e.g., Pinquart and Sörensen 2003, del-Pino-Casado, Palomino-Moral et al. 2017, Collins and Kishita 2019), though a causal relationship between these two variables cannot be inferred.

The examination of trajectories of depressive symptomatology for caregivers reporting different caregiving patterns, whether a single occasion, intermittent occasions, or two or more consecutive occasions, adds further nuance to these results. Similar to results reported by Lacey et al. (2019), our results suggest that caregivers who provide care intermittently and those who reported two or more consecutive care occasions experienced levels of depressive symptoms which remained relatively stable rather than decreased. This allows to disentangle whether the mean trajectory of depressive symptomatology was influenced by the single occasion caregivers, of which there were a majority in the study sample. In effect, these results suggest that the decrease in depressive symptomatology observed in the overall model was driven, at least in part, by the single occasion caregivers.

While the replication of previous results remains an important contribution the literature, particularly in a longitudinal context, a significant strength of the present study lies in its methodology. As described above, this study employs sophisticated statistical models to a large, cross-national sample of caregivers, something which has seldom been done due to the relative shortage of longitudinal data on caregivers. Compared to traditional approaches to longitudinal data analysis, growth curve modeling presents important advantages. Its strength and flexibility allow to accommodate for a variety of factors which often emerge in longitudinal analyses, such as partially missing data and the inclusion of time-varying covariates (Curran et al. 2010).

The present study is not without limitations. While SHARE’s moderate level of attrition is considered to be comparable to those of similar longitudinal studies (Börsch-Supan, Brandt et al. 2013, Bergmann, Kneip et al. 2019), it still poses a threat to the generalizability of this study’s findings. Further, certain challenges are inherent to the secondary analysis of data emerging from longitudinal studies on aging. Being that these studies tend to collect data on a very broad range of measures, the desired level of detail in a particular domain is not always available (Kaiser 2013). As such, certain variables which may be expected to have a significant impact on caregivers’ depressive symptoms over time could not be included in the present study. One such variable is the presence and quality of a supportive social network (Pearlin et al. 1990). SHARE introduced a module on social networks in its fourth and sixth waves of data collection (Börsch-Supan, Brandt et al. 2013), and it thus could not be included in the present study. Variables pertaining to caregiving intensity (e.g., number of hours spent caregiving, care recipient level of care dependence) might also be expected to influence the trajectory of depressive symptoms (Pearlin et al. 1990). While SHARE collects data on time spent caregiving, this variable was only collected for care provided outside the home (Börsch-Supan, Brandt et al. 2013) and was thus not included. Furthermore, the data do not provide information on the total span of caregiving in its participants. For instance, it is possible that a caregiver at Time 1 had been providing care consistently or intermittently for several years prior, which would be expected to influence the results as well. Finally, country- and region-specific factors such as the availability of formal supports play an important role in the outcomes experienced by caregivers (Wagner and Brandt 2018). While SHARE provides the opportunity to conduct cross-country comparison, it was beyond the scope of this study to do so in a meaningful way.

Despite these limitations, the results of this study provide valuable insights into the longitudinal course of depressive symptoms in a cross-national sample of caregivers over a significant period of time. Furthermore, this study opens numerous avenues for future research. One avenue is the replication of the present analysis on data emerging from existing or forthcoming longitudinal studies on aging to contribute to our understanding of the long-term impacts of caregiving on depressive symptomatology. Further, considering the significant within- and between-person differences identified in this study, future research may focus on determining whether discrete latent trajectories of depressive symptoms may be observed within the study sample.

## Conclusion

The results of the present study provide important insights into the pattern of depressive symptoms in a cross-national sample of caregivers over an extended period of time and contribute to the growing body of evidence on older caregivers’ mental health outcomes. This study has implications for improving our understanding of the long-term course of depressive symptoms among caregivers and in identifying caregivers who may experience poorer outcomes over time. Being that reliance on informal caregivers is steadily increasing and that care provision tends to be a dynamic and often long-term activity, continued efforts to understand the long-term implications of caregiving remain essential. As a vital segment of formal healthcare systems, this understanding may ensure that caregivers can maintain their roles and, perhaps most importantly, ensure the preservation of their mental health and well-being by identifying those individuals at greater risk of experiencing poorer outcomes (Bookman and Harrington 2007).

## Data Availability

This paper uses data from SHARE Waves 1, 2, 4, 5, 6, and 7 (DOIs: 10.6103/SHARE.w1.710, 10.6103/SHARE.w2.710, 10.6103/SHARE.w4.710, 10.6103/SHARE.w5.710, 10.6103/SHARE.w6.710, 10.6103/SHARE.w7.710) see Börsch-Supan et al. (2013) for methodological details.

## Appendix

See Tables 5, 6 and Fig. 5.

**Table 5:** Patterns of caregiving status at each measurement occasion

Caregiving status				
Time 1	Time 2	Time 3	Time 4	N
X				11,214
X	X			1748
X	X		X	60
X			X	195
X		X	X	49
X		X		357
X	X	X		293
X	X	X	X	71

Participants’ first instance of caregiving in the study was realigned to a new baseline (Time 1)

Data source: SHARE Release 7.1.0

**Table 6:** Fit indices for all latent growth curve models

Model	AIC	Adjusted BIC
*Mean trajectory with TVC*		
Linear	134,539.77	134,671.38
Quadratic	134,456.56	134,649.59
*Mean trajectory per pattern*		
Single occasion		
Linear	84,172.04	84,271.71
Quadratic	84,098.36	84,252.03
Intermittent		
Linear	9015.89	9047.86
Quadratic	9024.55	9073.85
Two or more occasions		
Linear	24,146.70	24,206.53
Quadratic	24,130.59	24,222.82

AIC = Akaike Information Criteria and Adjusted BIC = Sample Size Adjusted Bayesian Information Criteria. TVC = time-varying covariate. Data source: SHARE Release 7.1.0
